# Sex-specific effects of tea consumption and salt intake on metabolic syndrome and its components among oilfield workers

**DOI:** 10.3389/fnut.2025.1614417

**Published:** 2025-07-28

**Authors:** Haobiao Liu, Tianxiao Zhang, Lianxu Jia, Bowei Yang, Dianchao Zhang, Jing Han

**Affiliations:** ^1^Department of Health Management, Ningxia Gem Flower Hospital, Yinchuan, Ningxia, China; ^2^Department of Occupational and Environmental Health, School of Public Health, Health Science Center, Xi'an Jiaotong University, Xi'an, Shaanxi, China; ^3^Department of Epidemiology and Biostatistics, School of Public Health, Health Science Center, Xi'an Jiaotong University, Xi'an, Shaanxi, China; ^4^Department of Health Management, Xi'an Gem Flower Changqing Hospital, Xi'an, Shaanxi, China

**Keywords:** metabolic syndrome, tea consumption, salt intake, occupational health, cross-sectional study

## Abstract

**Objectives:**

The prevalence of metabolic syndrome (MetS) has been rising globally, posing a significant public health challenge. While numerous studies have examined the risk factors for MetS, limited research has explored the combined effects of tea consumption and salt intake on MetS, particularly from a sex-specific perspective.

**Methods:**

The Chinese Diabetes Society criteria were adopted to identify MetS patients, and the logistic regression analysis was employed to explore the association between tea consumption, salt intake, and MetS. The odds ratio (OR) and corresponding 95% confidence interval (CI) were calculated.

**Results:**

Among the 2,721 oilfield workers, 28.30% were diagnosed with MetS, with males displaying a higher prevalence than females across all individual components. Higher tea consumption was associated with an increased risk of MetS and specific components in males, whereas salt intake demonstrated a more selective impact, primarily affecting triglyceride levels in males and waist circumference in females. When considering the combined effects, males with high tea consumption and high salt intake exhibited the highest risk of MetS (OR = 1.83, 95% CI: 1.30 to 2.57, *p* < 0.001) compared to non-tea drinkers with low salt intake. However, no statistical significance was observed between different combinations of tea consumption and salt intake among females.

**Conclusion:**

This study highlights the sex-specific impact of tea consumption and salt intake on MetS, with a significant association observed only in males. The combined exposure to high tea consumption and high salt intake may exacerbate MetS risk, emphasizing the need for tailored dietary recommendations. Further prospective studies are warranted to confirm these findings.

## 1 Introduction

Metabolic syndrome (MetS), a cluster of metabolic abnormalities characterized by central obesity, dyslipidemia, hypertension, and hyperglycemia, poses a significant global health challenge (1). The prevalence of MetS has been steadily increasing worldwide, with approximately one-quarter of the adult population affected (2). This trend is evident in both developed and developing nations, necessitating urgent public health attention (3–6). In China, rapid economic development and lifestyle transitions have contributed to a sharp rise in MetS prevalence, escalating from 13.7% in 2001 to 31.1% between 2015 and 2017 (7, 8). This increase signals a growing epidemic that demands urgent attention, as individuals with MetS face higher risks of cardiovascular diseases, chronic kidney disease, and other obesity-related health issues (9, 10). Given the significant burden on healthcare systems, developing effective prevention and management strategies for MetS is imperative.

The etiology of MetS involves a complex interplay of genetic and environmental factors (8, 11, 12), with dietary habits emerging as critical lifestyle-related modifiable risk factors (8, 13). Among these, tea consumption and salt intake are two dietary behaviors widely prevalent in various cultures, including China. Certain studies have suggested that tea consumption may exert a protective effect against the development of MetS (14–17), potentially due to bioactive compounds such as polyphenols that have anti-inflammatory and antioxidant properties. Conversely, other research indicates a lack of significant association (18, 19) or even adverse effects (20, 21), particularly when tea is consumed in excess or combined with high-sugar additives. Similarly, excessive salt intake is known to contribute to the pathogenesis of MetS, particularly concerning hypertension and its association with obesity (22). However, the sex-specific effects of these dietary habits remain unclear, as existing studies often fail to adequately explore how these factors may differently influence males and females.

In certain regions of China, tea drinking is often culturally paired with salty foods, a pattern particularly notable among male workers in physically demanding occupations such as oilfield labor. Fieldwork environments with limited dietary options may reinforce the co-consumption of strong tea and high-sodium snacks or meals. Given this potential for interaction, it is important to examine the combined effects of tea and salt consumption on metabolic health in such populations.

Furthermore, occupational factors may further modify the relationship between diet and MetS. Oilfield workers, due to the physically demanding nature of their work, shift schedules, and unique dietary patterns, represent a population at heightened risk for metabolic disturbances. However, few studies have examined MetS in this occupational group, and the sex-specific effects of dietary exposures remain unexplored. Given the male-dominated nature of the oilfield workforce and the potential differential biological responses to dietary factors, investigating sex-specific associations is essential for targeted intervention strategies.

Considering these contextual factors, the present study aims to elucidate the sex-specific effects of tea consumption and salt intake on MetS and its components among oilfield workers. Our research will yield valuable information to inform public health policies and clinical practices, enabling tailored prevention strategies for oilfield workers and potentially other populations facing similar challenges. Ultimately, addressing the dietary contributors to MetS in this vulnerable workforce may significantly improve health outcomes and reduce MetS-associated disease burdens.

## 2 Materials and methods

### 2.1 Study design and participant recruitment

This cross-sectional study was conducted among oilfield workers undergoing routine occupational health examinations at a hospital in Xi’an, China, between October and December 2022. The target population comprised employees of a large oilfield enterprise, predominantly engaged in physically demanding manual labor, with the majority being male and aged between 30 and 60 years. Although detailed demographic data for the entire workforce were unavailable, the study sample is broadly representative of the occupational and demographic profile of this group.

Eligible participants were those aged 18 years or older, not pregnant, free from severe psychiatric disorders, and who voluntarily provided informed consent. Demographic information, lifestyle factors, and medical history were collected through face-to-face interviews using a standardized questionnaire administered by trained personnel. Anthropometric measurements and laboratory data were obtained from routine examination records.

A total of 4,121 individuals initially met the inclusion criteria. Of these, 292 lacked data on tea consumption, 291 had missing information on salt intake, 203 were missing diagnostic data for MetS, and 614 had incomplete covariate information, resulting in their exclusion. Ultimately, 2,721 participants were included in the final analysis (Supplementary Figure 1), yielding an effective inclusion rate of approximately 66.0%. To assess potential selection bias, baseline characteristics were compared between the initially eligible participants and those included in the analysis. To ensure adequate statistical power, the required sample size was calculated using a standard formula for cross-sectional studies:


$$N=\frac{Z_{1-\alpha /2}^{2}\mathrm{pq}}{d^{2}}$$

Where *Z* = 1.96 (corresponding to *α* = 0.05), *p* = 0.152 [the expected prevalence of MetS based on prior research (23)], *q* = 1–*p*, and d = 0.1 × *p* (allowable error). The minimum required sample size was calculated to be 2,144. After adjusting for an anticipated 20% non-response rate, the final required sample size was 2,573. The actual analytical sample of 2,721 participants exceeded this threshold.

### 2.2 Data measurement

Physical examination and biochemical testing were conducted by extensively trained technicians. Waist circumference (WC) was recorded using a tape measure at the level of the navel. Blood pressure (BP) was determined with a high-tech electronic sphygmomanometer (Omron HEM-7430) on the right upper limb after a 10-min rest period. All participants were requested to fast overnight for at least 8 h and to collect fasting blood samples the next morning for biochemical tests, including fasting blood glucose (FBG), triglycerides (TG), and high-density lipoprotein cholesterol (HDL-C).

### 2.3 Outcome assessment

Participants who had any three of the five following items were identified as MetS, based on the Chinese Diabetes Society criteria (24): (1) central obesity: WC ≥ 90 cm in males or ≥85 cm in females. (2) Hyperglycemia: FPG ≥ 6.1 mmol/L, 2-h plasma glucose ≥7.8 mmol/L, or previously diagnosed with diabetes mellitus. (3) High BP: systolic blood pressure (SBP) ≥ 130 mmHg, diastolic blood pressure (DBP) ≥ 85 mmHg, or confirmed and treated for hypertension. (4) Low HDL-C: serum HDL-C < 1.04 mmol/L. (5) High TG: TG ≥ 1.7 mmol/L.

### 2.4 Exposure ascertainment

Tea drinking information was collected through two questions: “How many days a week do you drink tea?” and “How many milliliters (mL) of tea do you drink each day?.” To improve the accuracy of estimation, trained investigators presented a standard cup (250 mL) on-site as a visual reference. Participants were instructed to use this cup as a guide when estimating their daily tea intake, thereby minimizing variability due to differences in individual cup size perceptions. Weekly tea consumption was calculated by multiplying the reported frequency (days/week) by the daily amount (mL/day). Based on this, participants were categorized into non-tea drinkers (0 mL/week) and tea drinkers. Among tea drinkers, consumption was further classified into low (<2,250 mL/week) and high (≥2,250 mL/week) based on the median intake.

Salt intake information was gathered from the questionnaire item “What is your average daily salt intake?” with the alternative options of ≤6 g/day, defined as low salt intake, and >6 g/day, defined as high salt intake. To enhance the accuracy of classification, a standard tablespoon was displayed to participants, and they were informed that one tablespoon approximately equals 6 grams of salt. This visual reference assisted participants in estimating their usual intake level.

### 2.5 Covariate definition

The potential covariates were selected as follows: age, ethnicity, education level, marital status, annual income, shift work, chemical substance exposure, noise exposure, dust exposure, cigarette smoking, alcohol drinking, physical activity, and food diversity. Detailed information regarding covariates can be found in Supplementary Table 1.

### 2.6 Statistical analysis

Continuous variables are presented as means (standard deviation, SD) or medians (interquartile range, IQR), depending on whether they meet the criteria for normal distribution. A *t*-test or non-parametric test was performed to compare differences between groups as appropriate. Categorical variables are expressed as numbers (percentages), and a chi-square test was conducted for group comparisons. Logistic regression analysis was employed to explore potential factors associated with the outcomes, with results presented as odds ratios (OR) and corresponding 95% confidence intervals (CI). To assess potential effect modification by sex, interaction terms were included in the multivariable logistic regression models. Furthermore, sensitivity analyses were conducted to test the robustness of the findings, which included employing imputation for covariates and performing analyses based on National Cholesterol Education Program Adult Treatment Panel III criteria for MetS. All analyses and visualizations were carried out using R (version 4.4.0). A two-sided *p* value <0.05 was considered statistically significant.

## 3 Results

### 3.1 Characteristic features of participants

The basic characteristics of the participants are listed in Table 1. The mean age of participants was 41.10 years, with 36.24% identifying as female and the majority (98.02%) being of Han ethnicity. Most participants were married (83.24%), and 65.56% had an annual income between 101,000 and 150,000 CNY. Compared to non-MetS respondents, those diagnosed with MetS were more likely to be older and male (*p* < 0.001). They also had lower educational levels and were more prone to smoking and drinking (*p* < 0.001). Furthermore, individuals with MetS had higher levels of tea consumption (*p* < 0.001) and salt intake (*p* = 0.002) than those without MetS. No statistical differences were observed in ethnicity, annual income, physical activity, or food diversity (*p* > 0.05). Notably, significant differences were also found in age, sex, annual income, smoking, and alcohol drinking (*p* < 0.05) between the initially eligible participants with those included in the final analysis (Supplementary Table 2).

**Table 1:** Basic characteristics of participants.

Variables	Total (*N* = 2,721)	Non-MetS (*N* = 1,951)	MetS (*N* = 770)	*P*-value
Age, year	41.10 (8.38)	40.39 (8.30)	42.90 (8.34)	<0.001
Sex				<0.001
Male	1735 (63.76)	1,056 (54.13)	679 (88.18)	
Female	986 (36.24)	895 (45.87)	91 (11.82)	
Ethnicity				0.498
Han	2,667 (98.02)	1915 (98.15)	752 (97.66)	
Other	54 (1.98)	36 (1.85)	18 (2.34)	
Education level				<0.001
High school or below	981 (36.05)	658 (33.73)	323 (41.95)	
College degree	952 (34.99)	683 (35.01)	269 (34.94)	
University graduate or above	788 (28.96)	610 (31.27)	178 (23.12)	
Marital status				0.006
Unmarried	326 (11.98)	258 (13.22)	68 (8.83)	
Married	2,265 (83.24)	1,601 (82.06)	664 (86.23)	
Separated	130 (4.78)	92 (4.72)	38 (4.94)	
Annual income, thousand (CNY)				0.381
≤100	534 (19.63)	393 (20.14)	141 (18.31)	
101–150	1784 (65.56)	1,278 (65.50)	506 (65.71)	
≥151	403 (14.81)	280 (14.35)	123 (15.97)	
Height, m	169.40 (8.13)	168.18 (8.22)	172.51 (7.02)	<0.001
Weight, Kg	70.67 (13.96)	66.33 (12.09)	81.65 (12.25)	<0.001
Body mass index, Kg/m^2^	24.49 (3.75)	23.35 (3.24)	27.39 (3.37)	<0.001
Shift work				0.038
No	1,171 (43.04)	815 (41.77)	356 (46.23)	
Yes	1,550 (56.96)	1,136 (58.23)	414 (53.77)	
Chemical substance exposure				0.914
No	585 (21.50)	421 (21.58)	164 (21.30)	
Yes	2,136 (78.50)	1,530 (78.42)	606 (78.70)	
Noise exposure				0.536
No	1,051 (38.63)	746 (38.24)	305 (39.61)	
Yes	1,670 (61.37)	1,205 (61.76)	465 (60.39)	
Dust exposure				0.317
No	2083 (76.55)	1,504 (77.09)	579 (75.19)	
Yes	638 (23.45)	447 (22.91)	191 (24.81%)	
Cigarette smoking				<0.001
No	1,513 (55.60)	1,246 (63.86)	267 (34.68)	
Yes	1,208 (44.40)	705 (36.14)	503 (65.32)	
Alcohol drinking				<0.001
No	1777 (65.31)	1,393 (71.40)	384 (49.87)	
Yes	944 (34.69)	558 (28.60)	386 (50.13)	
Physical activity				0.209
Low	1,050 (38.59)	769 (39.42)	281 (36.49)	
Medium	799 (29.36)	575 (29.47)	224 (29.09)	
High	872 (32.05)	607 (31.11)	265 (34.42)	
Food diversity				0.843
<4 types/day	1,347 (49.50)	963 (49.36)	384 (49.87)	
≥4 types/day	1,374 (50.50)	988 (50.64)	386 (50.13)	
Tea consumption				<0.001
None	1,445 (53.11)	1,134 (58.12)	311 (40.39)	
Low	655 (24.07)	458 (23.48)	197 (25.58)	
High	621 (22.82)	359 (18.40)	262 (34.03)	
Salt intake				0.002
Low	1,326 (48.73)	988 (50.64)	338 (43.90)	
High	1,395 (51.27)	963 (49.36)	432 (56.10)	

MetS, metabolic syndrome; SD, standard deviation; CNY, Chinese Yuan.

Regarding tea consumption levels, participants with higher tea consumption tended to have a greater prevalence of three specific MetS components (*p* < 0.05), except for high TG (*p* = 0.069) and low HDL-C (*p* = 0.131), among male individuals. They also exhibited higher levels of the corresponding indicators (*p* < 0.05), except for TG (*p* = 0.154). However, statistical significance was detected only in SBP (*p* = 0.014) among females (Supplementary Table 3). In terms of salt intake, only the elevated WC (*p* = 0.048) and high TG (*p* = 0.016) components, as well as the indicator TG (*p* = 0.009) among male respondents demonstrated statistical significance at high salt intake levels compared to those with low salt intake. A similar pattern of elevated WC component (*p* = 0.025) and WC value (*p* = 0.005) was also observed among female participants (Supplementary Table 4).

### 3.2 Prevalence of MetS and its components

**Figure 1:**
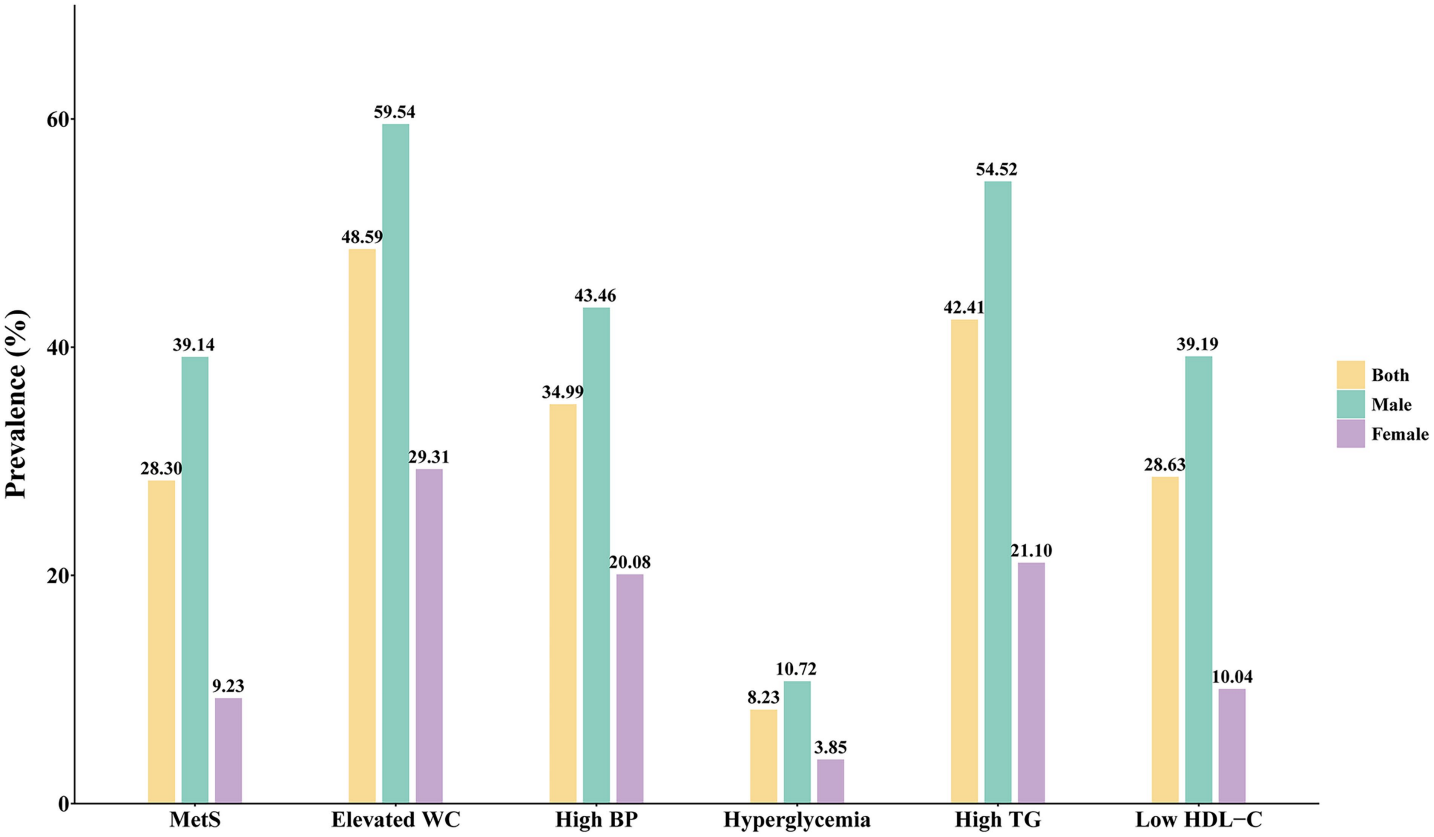
Prevalence of metabolic syndrome and its components. MetS, metabolic syndrome; WC, waist circumference; BP, blood pressure; TG, triglycerides; HDL-C, high-density lipoprotein cholesterol.

Among the 2,721 subjects analyzed, 770 (28.30%) were clinically diagnosed with MetS (Figure 1). The prevalence of MetS varied by sex, with rates of 39.14% in males and 9.23% in females, indicating a pronounced sex difference. Regarding individual components of MetS, elevated WC exhibited the highest prevalence at 48.59%, followed closely by high TG at 42.41%. In contrast, hyperglycemia was observed in only 8.23% of the participants, making it the least prevalent component across the entire study population. Similar patterns were noted when analyzing these components by sex; notably, males consistently displayed higher prevalence rates than females across all individual components.

### 3.3 Association between tea consumption and MetS and its components

The independent association between tea consumption and MetS and its components was analyzed separately for males and females (Table 2). In male participants, the crude model showed that high tea consumption was significantly associated with a higher risk of MetS (OR = 1.78, 95% CI: 1.41 to 2.24, *p* < 0.001) compared to non-tea drinkers. Among the individual MetS components, high tea consumption also significantly associated with all components, with an increased risk of 1.54 times for elevated WC (OR = 1.54, 95% CI: 1.22 to 1.94, *p* < 0.001), 1.48 times for high BP (OR = 1.48, 95% CI: 1.18 to 1.85, *p* < 0.001), 2.27 times for hyperglycemia (OR = 2.27, 95% CI: 1.58 to 3.28, *p* < 0.001), 1.30 times for high TG (OR = 1.30, 95% CI: 1.04 to 1.63, *p* = 0.022), and 1.26 times for low HDL-C (OR = 1.26, 95% CI: 1.00 to 1.59, *p* = 0.046) when compared to those without tea-drinking. Low tea consumption was also associated with a 31% increased risk of elevated WC (OR = 1.31, 95% CI: 1.03 to 1.65, *p* = 0.026). After multivariable adjustment, high tea consumption was significantly associated with a 45% increased risk of MetS (OR = 1.45, 95% CI: 1.13 to 1.86, *p* = 0.003) in males compared to non-tea drinkers. Among MetS components, the risk of elevated WC increased by 48% (OR = 1.48, 95% CI: 1.16 to 1.90, *p* = 0.002), and the risk of hyperglycemia increased by 62% (OR = 1.62, 95% CI: 1.10 to 2.40, *p* = 0.014) for the high tea group. The risk of elevated WC is still associated with low tea consumption in male participants, with an increased risk of 1.29 times (OR = 1.29, 95% CI: 1.01 to 1.66, *p* = 0.040) when compared to the non-tea drinkers. No statistically significant associations were found for high BP, high TG, or low HDL-C in the adjusted model. In contrast, no associations were observed among females, suggesting a sex-specific pattern.

**Table 2:** Association between tea consumption and metabolic syndrome and its components.

Component	Tea consumption	Male				Female			
		Crude model		Adjusted model		Crude model		Adjusted model	
		OR (95% CI)	*P*-value	OR (95% CI)	*P*-value	OR (95% CI)	*P*-value	OR (95% CI)	*P*-value
MetS									
	None	1.00 (Reference)		1.00 (Reference)		1.00 (Reference)		1.00 (Reference)	
	Low	1.21 (0.95, 1.53)	0.125	1.05 (0.81, 1.35)	0.705	0.93 (0.50, 1.63)	0.802	0.86 (0.46, 1.54)	0.628
	High	**1.78 (1.41, 2.24)**	**<0.001**	**1.45 (1.13, 1.86)**	**0.003**	1.34 (0.65, 2.56)	0.395	1.16 (0.54, 2.29)	0.686
Elevated WC									
	None	1.00 (Reference)		1.00 (Reference)		1.00 (Reference)		1.00 (Reference)	
	Low	**1.31 (1.03, 1.65)**	**0.026**	**1.29 (1.01, 1.66)**	**0.040**	0.98 (0.67, 1.40)	0.893	1.01 (0.69, 1.46)	0.973
	High	**1.54 (1.22, 1.94)**	**<0.001**	**1.48 (1.16, 1.90)**	**0.002**	1.04 (0.64, 1.65)	0.877	1.07 (0.64, 1.74)	0.801
High BP									
	None	1.00 (Reference)		1.00 (Reference)		1.00 (Reference)		1.00 (Reference)	
	Low	1.24 (0.98, 1.57)	0.070	0.99 (0.77, 1.27)	0.933	0.89 (0.58, 1.35)	0.599	0.79 (0.50, 1.22)	0.294
	High	**1.48 (1.18, 1.85)**	**<0.001**	1.11 (0.87, 1.42)	0.407	1.41 (0.84, 2.29)	0.182	1.22 (0.70, 2.08)	0.464
Hyperglycemia									
	None	1.00 (Reference)		1.00 (Reference)		1.00 (Reference)		1.00 (Reference)	
	Low	1.49 (1.00, 2.23)	0.051	1.21 (0.79, 1.83)	0.384	2.09 (0.96, 4.31)	0.053	2.01 (0.87, 4.39)	0.087
	High	**2.27 (1.58, 3.28)**	**<0.001**	**1.62 (1.10, 2.40)**	**0.014**	1.79 (0.59, 4.51)	0.252	1.35 (0.41, 3.67)	0.588
High TG									
	None	1.00 (Reference)		1.00 (Reference)		1.00 (Reference)		1.00 (Reference)	
	Low	1.08 (0.86, 1.37)	0.500	0.97 (0.76, 1.24)	0.818	1.08 (0.71, 1.60)	0.720	1.02 (0.66, 1.55)	0.919
	High	**1.30 (1.04, 1.63)**	**0.022**	1.12 (0.88, 1.43)	0.366	1.45 (0.87, 2.35)	0.142	1.32 (0.76, 2.22)	0.311
Low HDL-C									
	None	1.00 (Reference)		1.00 (Reference)		1.00 (Reference)		1.00 (Reference)	
	Low	1.07 (0.85, 1.36)	0.560	1.06 (0.82, 1.36)	0.647	0.58 (0.29, 1.05)	0.089	0.56 (0.28, 1.04)	0.085
	High	**1.26 (1.00, 1.59)**	**0.046**	1.18 (0.92, 1.51)	0.192	0.65 (0.26, 1.36)	0.290	0.60 (0.23, 1.32)	0.244

Crude model, no covariate was adjusted. Adjusted model, adjusted for age, ethnicity, education level, marital status, annual income, shift work, chemical substance exposure, noise exposure, dust exposure, cigarette smoking, alcohol drinking, physical activity, and food diversity. Results in bold indicate statistical significance. OR, odds ratio; CI, confidence interval; MetS, metabolic syndrome; WC, waist circumference; BP, blood pressure; TG, triglycerides; HDL-C, high-density lipoprotein cholesterol.

### 3.4 Association between salt intake and MetS and its components

The association between salt intake and MetS and its components was also analyzed separately by sex (Table 3). In males, high salt intake was not significantly associated with overall MetS risk in the crude model (OR = 1.17, 95% CI: 0.96 to 1.42, *p* = 0.113). However, significant positive associations were observed for individual components. It’s noted that high salt intake was positively associated with elevated WC (OR = 1.22, 95% CI: 1.01 to 1.48, *p* = 0.043) and high TG (OR = 1.27, 95% CI: 1.05 to 1.54, *p* = 0.014). No significant associations were observed between salt intake and the other three components. After adjusting for potential confounders, the association between salt intake and MetS remained non-significant in both sexes. Nonetheless, in males, a 25% increased risk of high TG was still observed with high salt intake (OR = 1.25, 95% CI: 1.02 to 1.52, *p* = 0.029). In females, salt intake was not associated with MetS overall. However, female participants with high salt intake had a 40% increased risk of central obesity, with elevated WC (OR = 1.40, 95% CI: 1.15 to 1.86, *p* = 0.022) compared to those with lower intake. These findings suggest that salt intake may influence specific components of MetS in a sex-specific manner, particularly affecting lipid metabolism in males and fat distribution in females.

**Table 3:** Association between salt intake and metabolic syndrome and its components.

Component	Salt intake	Male				Female			
		Crude model		Adjusted model		Crude model		Adjusted model	
		OR (95% CI)	*P*-value	OR (95% CI)	*P*-value	OR (95% CI)	*P*-value	OR (95% CI)	*P*-value
MetS									
	Low	1.00 (Reference)		1.00 (Reference)		1.00 (Reference)		1.00 (Reference)	
	High	1.17 (0.96, 1.42)	0.113	1.19 (0.98, 1.46)	0.085	1.11 (0.72, 1.72)	0.626	1.23 (0.79, 1.93)	0.361
Elevated WC									
	Low	1.00 (Reference)		1.00 (Reference)		1.00 (Reference)		1.00 (Reference)	
	High	**1.22 (1.01, 1.48)**	**0.043**	1.20 (0.98, 1.46)	0.075	**1.38 (1.05, 1.82)**	**0.021**	**1.40 (1.05, 1.86)**	**0.022**
High BP									
	Low	1.00 (Reference)		1.00 (Reference)		1.00 (Reference)		1.00 (Reference)	
	High	1.03 (0.85, 1.24)	0.782	1.08 (0.88, 1.32)	0.475	0.78 (0.56, 1.07)	0.120	0.79 (0.57, 1.11)	0.183
Hyperglycemia									
	Low	1.00 (Reference)		1.00 (Reference)		1.00 (Reference)		1.00 (Reference)	
	High	1.07 (0.79, 1.46)	0.659	1.21 (0.88, 1.66)	0.246	1.24 (0.65, 2.39)	0.515	1.26 (0.63, 2.51)	0.510
High TG									
	Low	1.00 (Reference)		1.00 (Reference)		1.00 (Reference)		1.00 (Reference)	
	High	**1.27 (1.05, 1.54)**	**0.014**	**1.25 (1.02, 1.52)**	**0.029**	1.12 (0.83, 1.53)	0.456	1.15 (0.83, 1.59)	0.389
Low HDL-C									
	Low	1.00 (Reference)		1.00 (Reference)		1.00 (Reference)		1.00 (Reference)	
	High	1.00 (0.82, 1.21)	0.960	1.02 (0.84, 1.25)	0.827	0.90 (0.59, 1.36)	0.613	0.90 (0.58, 1.40)	0.657

Crude model, no covariate was adjusted. Adjusted model, adjusted for age, ethnicity, education level, marital status, annual income, shift work, chemical substance exposure, noise exposure, dust exposure, cigarette smoking, alcohol drinking, physical activity, and food diversity. Results in bold indicate statistical significance. OR, odds ratio; CI, confidence interval; MetS, metabolic syndrome; WC, waist circumference; BP, blood pressure; TG, triglycerides; HDL-C, high-density lipoprotein cholesterol.

### 3.5 Combined effects of tea consumption and salt intake on MetS and its components

**Figure 2:**
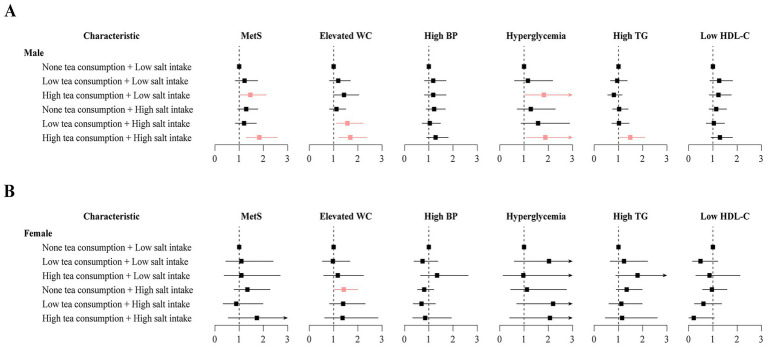
Sex-specific associations between combined tea consumption and salt intake with metabolic syndrome and its components among oilfield workers. **(A)** Male; **(B)** female. Multivariable logistic regression models were adjusted for age, ethnicity, education level, marital status, annual income, shift work, chemical substance exposure, noise exposure, dust exposure, cigarette smoking, alcohol drinking, physical activity, and food diversity. The color red represents statistical significance. Tea consumption was calculated as weekly volume (mL/week) based on reported frequency (days/week) and average daily intake (mL/day), and categorized into none (0 mL/week), low (<2,250 mL/week), and high (≥2,250 mL/week). Salt intake was self-reported and categorized as low (≤6 g/day) or high (>6 g/day). MetS, metabolic syndrome; WC, waist circumference; BP, blood pressure; TG, triglycerides; HDL-C, high-density lipoprotein cholesterol.

When analyzing the combined effects of tea consumption and salt intake, male participants with high tea consumption and low salt intake had a 46% higher odds of MetS (OR = 1.46, 95% CI: 1.02 to 2.11, *p* = 0.040), and those with both high tea and high salt intake had an 83% higher odds of MetS (OR = 1.83, 95% CI: 1.30 to 2.57, *p* < 0.001), compared to those with no tea consumption and low salt intake. The highest risk was observed in the high tea and high salt group, indicating a potential synergistic adverse effect (Figure 2). In contrast, no statistically significant associations were observed in female participants across any combined exposure patterns (Supplementary Table 5).

For MetS components among male participants, combined tea and salt intake patterns showed specific associations. Compared to those with no tea and low salt intake, males with low tea consumption and high salt intake had a 57% higher odds of elevated WC (OR = 1.57, 95% CI: 1.11 to 2.22, *p* = 0.011). In addition, those with high tea consumption and low salt intake exhibited an 82% higher odds of hyperglycemia (OR = 1.82, 95% CI: 1.03 to 3.26, *p* = 0.040). Those in the high tea consumption and high salt intake group exhibited 69% higher risk of central obesity (OR = 1.69, 95% CI: 1.21 to 2.37, *p* = 0.002), 88% higher risk of hyperglycemia (OR = 1.88, 95% CI: 1.10 to 3.29, *p* = 0.024), and 49% higher risk of high TG (OR = 1.49, 95% CI: 1.06 to 2.09, *p* = 0.020). None tea consumption and high salt intake group also demonstrated greater odds of elevated WC (OR = 1.42, 95% CI: 1.02 to 1.99, *p* = 0.040) among female individuals (Supplementary Table 5).

Although stratified analyses suggested stronger associations in males, formal interaction tests were not statistically significant (*P* for interaction > 0.05), indicating that sex did not significantly modify the association between dietary exposures and MetS and its components (Supplementary Table 6).

### 3.6 Sensitivity analysis

Sensitivity analysis further confirmed the robustness of our findings. After employing imputation for covariates, a comparative association was also revealed between high tea consumption and low salt intake (OR = 1.43, 95% CI: 1.04 to 1.96, *p* = 0.028), as well as between high tea consumption and high salt intake (OR = 1.84, 95% CI: 1.37 to 2.47, *p* < 0.001) with MetS among male participants. However, no statistical significance was observed among females (Supplementary Table 7). When performing analyses based on the National Cholesterol Education Program Adult Treatment Panel III criteria for MetS, a similar pattern emerged, with an elevated risk of MetS detected in high tea consumption and low salt intake (OR = 1.51, 95% CI: 1.06 to 2.16, *p* = 0.022), as well as in high tea consumption and high salt intake (OR = 1.96, 95% CI: 1.41 to 2.73, *p* < 0.001) among male participants (Supplementary Table 8).

## 4 Discussion

This study provides novel insights into the sex-specific effects of tea consumption and salt intake on MetS and its components among oilfield workers. Our findings reveal an intricate relationship between these dietary factors and MetS risk, with significant differences observed between male and female participants. Notably, high tea consumption was associated with an increased risk of MetS and its components in males, whereas salt intake demonstrated a more selective impact, primarily affecting TG levels in males and WC in females. Furthermore, the combined effects of high tea consumption and high salt intake were particularly pronounced in males, suggesting a possible synergistic contribution to metabolic dysfunction.

Contrary to some previous studies suggesting a protective effect of tea on metabolic health, our findings indicate that high tea consumption is positively associated with MetS among male oilfield workers. Specifically, high tea consumption was linked to increased odds of central obesity, hyperglycemia, and, to a lesser extent, dyslipidemia and hypertension. Similar findings have also been reported in prior studies, supporting the plausibility of our results. A prospective cohort study in rural northeastern China found that drinking tea 1–2 times per day increased the incidence of MetS by 37.6% (25). Another community-based cohort study reported that individuals who drank tea more than five times per week experienced a higher cumulative incidence of MetS (OR = 1.38) compared with non-habitual tea drinkers (21). The unexpected positive association between high tea consumption and MetS in males may be partially explained by unmeasured factors, such as the consumption of sugar-sweetened tea or tea mixed with milk, both of which are common in some regions and may contribute to increased caloric and sugar intake. Unfortunately, our data did not include information on tea type or additives, which limits mechanistic interpretation. Further, it is possible that sex-specific behaviors—such as male preference for stronger or sweetened tea, or concurrent high-salt food intake during tea drinking—may modify metabolic responses. The lack of significant findings in females may be attributed to differences in metabolic responses, hormonal regulation, or lifestyle factors, warranting further investigation into sex-specific physiological mechanisms.

The role of salt intake in MetS development has been well-documented, primarily through its impact on hypertension. However, our study did not find a direct association between high salt intake and MetS in either sex. Instead, high salt intake was specifically associated with elevated TG levels in males and increased WC in females. The link between salt intake and dyslipidemia in males is consistent with emerging evidence suggesting that excessive sodium intake may impair lipid metabolism and promote adipogenesis (26). Meanwhile, the observed relationship between salt intake and central obesity in females aligns with prior findings indicating sex-specific sodium handling and fluid retention patterns (22). These results highlight the need for tailored dietary recommendations that account for sex-based metabolic differences.

A key finding of our study is the combined effect of high tea consumption and high salt intake on the risk of MetS, particularly among males. Compared to non-tea drinkers with low salt intake, males who consumed both high amounts of tea and salt exhibited the highest odds of MetS and its components, including elevated WC, hyperglycemia, and high TG. These cooperative influences suggest that these dietary factors may work together to exacerbate metabolic dysfunction. However, limited studies have examined the combined effects of tea consumption and salt intake, particularly in a sex-specific manner, highlighting the novelty of our study. Notably, a cross-sectional survey conducted in the Tibetan highland regions revealed that hypertensive patients were more likely to drink tea with added salt compared to normotensive subjects (27). Those who consumed tea with salt had a 1.33-fold increased risk of developing hypertension, underscoring the potential adverse effects of combining tea and salt intake. Our findings suggest that when high tea consumption is combined with high salt intake, its adverse effects on metabolism may be amplified. Furthermore, the observed sex-specific differences may be related to hormonal or metabolic variations between males and females. Previous studies have indicated that estrogen may play a protective role in metabolic regulation (28), potentially mitigating the negative effects of dietary factors on MetS risk in females. The smaller number of female participants and their lower MetS prevalence may have limited the statistical power to detect associations in females. Future studies with larger female cohorts and longitudinal designs are needed to confirm these findings. Notably, the observed sex differences in stratified analyses did not reach statistical significance in formal interaction tests, possibly due to limited statistical power. Nonetheless, the sex-specific patterns may still have practical relevance and should be explored further in larger studies.

Oilfield workers often face unique occupational challenges such as shift work, irregular meal schedules, high physical demands, and reliance on high-sodium convenience foods. Additionally, in certain regions of China, strong tea consumption is culturally ingrained and often accompanied by salty snacks, which may compound metabolic risks. These patterns may be especially pronounced in oilfield workers due to constrained food choices during shift work or field deployment. The cultural practice of drinking strong tea with savory foods warrants further investigation as a potential modifier of metabolic risk. Moreover, tea consumption in this population may act as a proxy for other unmeasured risk factors. For example, sweetened or concentrated tea may reflect higher sugar intake or be consumed with calorie-dense snacks. Similarly, high salt intake may signal a broader dietary pattern rich in processed or preserved foods. These patterns underscore the need for future research using more detailed dietary assessments and objective biomarkers.

The underlying mechanisms linking tea consumption and salt intake to MetS are complex and multifactorial. Tea contains various bioactive components, including tea polyphenols, tea polysaccharides, L-theanine, tea pigments, caffeine, and tea saponins, which can exert both beneficial and harmful metabolic effects (29, 30). Excessive intake may have pro-oxidant effects, generating reactive oxygen species that damage pancreatic β-cells and induce insulin resistance (31). Tea components may also disrupt gut microbiota, interfere with iron absorption, and negatively impact metabolic homeostasis (32). High salt intake, in turn, can exacerbate oxidative stress, increase sympathetic nervous system activity, and contribute to endothelial dysfunction (33). It may also reduce the bioavailability of tea polyphenols, potentially diminishing their protective effects. In addition, salt-induced fluid retention and elevated blood pressure may further aggravate caffeine-related sympathetic activation (34). Collectively, these potential mechanisms may contribute to the increased risk of developing MetS among individuals with high tea consumption and salt intake.

Our study underscores the importance of considering sex-specific dietary effects in MetS prevention strategies, particularly in high-risk occupational populations such as oilfield workers. Given the demanding nature of their work and potential dietary imbalances, targeted interventions are needed to promote healthier consumption patterns. Reducing excessive tea consumption—especially in combination with high salt intake—may be an effective strategy for mitigating MetS risk in male workers. Meanwhile, efforts to curb salt intake may be particularly beneficial for addressing sex-specific metabolic concerns, such as TG elevations in males and central obesity in females.

Although this study has several strengths, certain limitations should be acknowledged. First, its cross-sectional design precludes causal inferences, and longitudinal studies are needed to establish temporal relationships. Second, tea and salt intake were self-reported and thus susceptible to recall bias. While standardized visual aids were used to enhance estimation accuracy, measurement error remains possible. Third, data collection was conducted during the winter season, when tea consumption may increase, potentially leading to an overestimation of habitual intake. Additionally, information on tea type (e.g., green, black, oolong) and seasonal variation in consumption was not collected, which may limit the interpretation of findings, given the differing metabolic effects of various tea types. Fourth, baseline characteristics differed significantly between participants included in the final analysis and those initially eligible, particularly with respect to age, sex, income, smoking, and alcohol consumption, suggesting potential selection bias that may affect the generalizability of the results. Finally, despite adjustment for multiple covariates, residual confounding from unmeasured lifestyle or genetic factors cannot be ruled out. Future studies should incorporate objective biomarkers and investigate underlying mechanisms to further clarify these associations.

## 5 Conclusion

In conclusion, this study highlights the sex-specific effects of tea consumption and salt intake on MetS risk among oilfield workers. High tea consumption was associated with an increased risk of MetS and its components in males but not in females, while high salt intake demonstrated selective metabolic effects, including elevated TG in males and increased central obesity in females. Notably, the combination of high tea and high salt intake further exacerbated MetS risk, particularly among males, suggesting potential dietary interactions that warrant further investigation. These findings underscore the need for sex-specific dietary interventions in occupational health settings. Public health strategies targeting this high-risk population should emphasize moderation in tea intake, discourage the consumption of sweetened or salty tea accompaniments, and promote reduced salt consumption. Tailored workplace nutrition programs and regular health screenings may play a pivotal role in mitigating cardiometabolic risks among vulnerable working populations.

## Data Availability

The raw data supporting the conclusions of this article will be made available by the authors, without undue reservation.
