# Supplementary material for: Sex-specific effects of tea consumption and salt intake on metabolic syndrome and its components among oilfield workers
Source: Front Nutr. 2025 Jul 28;12:1614417. doi: 10.3389/fnut.2025.1614417 (PMC12336240; doi:10.3389/fnut.2025.1614417)
Supplement: Supplementary file 1 [file Data_Sheet_1.pdf]

Supplementary Material

Table S1. Category and definition of covariates.

Table S2. Comparison of baseline characteristics between initially eligible participants and those included in the final analysis

Table S3. Basic characteristics of participants by tea consumption level.

Table S4. Basic characteristics of participants by salt intake level.

Table S5. Association of tea consumption and salt intake on metabolic syndrome and its components.

Table S6. Results of interaction analyses between sex and tea consumption, salt intake, and their combined exposure on metabolic syndrome and its components.

Table S7. Association of tea consumption and salt intake on metabolic syndrome and its components, after employing imputation for covariates.

Table S8. Association of tea consumption and salt intake on metabolic syndrome and its components, performing analyses based on National Cholesterol Education Program Adult Treatment Panel III criteria for MetS.

Figure S1. Flow diagram of participants included in this study.

Table S1. Category and definition of covariates.

Variable	Category and definition
Shift work	Shift work is defined as the regular rotation of individuals to work outside the hours of 8:00 AM to 5:00 PM for a minimum duration of one year.
Chemical substance exposure	Chemical substance exposure is defined as the self-reported exposure of individuals to hazardous chemical substances in the workplace or environment, including benzene, toluene, xylene, hydrogen sulfide, carbon monoxide, nitrogen oxides, carbon tetrachloride, n-hexane, n-pentane, gasoline, etc.
Noise exposure	Noise exposure is defined as the self-reported exposure of individuals to noise levels in the work environment that exceed legal noise standards.
Dust exposure	Dust exposure is defined as the self-reported exposure of individuals to inhalable or respirable dust particles during the work process, which may originate from production processes, material handling, or the surrounding environment.
Cigarette smoking	Cigarette smokers are defined as individuals who smoke at least one cigarette daily for six months or longer.
Alcohol drinking	Alcohol drinkers are classified as those who consume alcohol at least once a week and maintain this frequency for six months or longer.
Physical activity	Physical activity is calculated by multiplying the corresponding frequency (days/week) and duration (minutes/day) of moderate activity. Vigorous activity is transformed into double the amount of moderate activity to standardize the calculation. Physical activity levels are then categorized into low (<150 min/week), medium (151–<600 min/week), and high (≥600 min/week).
Food diversity	Food diversity is defined by the self-reported number of different food types consumed daily and categorized as <4 types/day and ≥4 types/day.

Table S2. Comparison of baseline characteristics between initially eligible participants and those included in the final analysis

Variables	Total recruited	Final analyzed	*P* value
**Age, year**	41.66 (8.42)	41.10 (8.38)	0.007
**Sex**			0.012
Male	2751 (66.76)	1735 (63.76)	
Female	1370 (33.24)	986 (36.24)	
**Ethnicity**			0.679
Han	3845 (98.19)	2667 (98.02)	
Other	71 (1.81)	54 (1.98)	
**Education level**			0.059
High school or below	1495 (38.61)	981 (36.05)	
College degree	1341 (34.63)	952 (34.99)	
University graduate or above	1036 (26.76)	788 (28.96)	
**Marital status**			0.658
Unmarried	429 (11.25)	326 (11.98)	
Married	3200 (83.90)	2265 (83.24)	
Separated	185 (4.85)	130 (4.78)	
**Annual income, thousand (CNY)**			0.014
≤100	839 (22.38)	534 (19.63)	
101-150	2335 (62.28)	1784 (65.56)	
≥151	575 (15.34)	403 (14.81)	
**Height, m**	169.59 (7.95)	169.40 (8.13)	0.351
**Weight, Kg**	70.99 (13.73)	70.67 (13.96)	0.351
**Body mass index, Kg/m^2**	24.56 (3.73)	24.49 (3.75)	0.462
**Shift work**			0.523
No	1703 (43.86)	1171 (43.04)	
Yes	2180 (56.14)	1550 (56.96)	
**Chemical substance exposure**			0.053
No	925 (23.55)	585 (21.50)	
Yes	3002 (76.45)	2136 (78.50)	
**Noise exposure**			0.405
No	1456 (37.58)	1051 (38.63)	
Yes	2418 (62.42)	1670 (61.37)	
**Dust exposure**			0.075
No	2840 (74.60)	2083 (76.55)	
Yes	967 (25.40)	638 (23.45)	
**Cigarette smoking**			0.003
No	2017 (51.82)	1513 (55.60)	
Yes	1875 (48.18)	1208 (44.40)	
**Alcohol drinking**			0.044
No	2420 (62.86)	1777 (65.31)	

Yes	1430 (37.14)	944 (34.69)	
**Physical activity**			0.876
Low	1425 (38.23)	1050 (38.59)	
Medium	1085 (29.11)	799 (29.36)	
High	1217 (32.65)	872 (32.05)	
**Food diversity**			0.926
<4 types/day	1916 (49.36)	1347 (49.50)	
≥4 types/day	1966 (50.64)	1374 (50.50)	

MetS, metabolic syndrome; SD, standard deviation; CNY, China Yuan.

Table S3. Basic characteristics of participants by tea consumption level.

Sex	Component/Indicator	Tea consumption level			*P* value
		None	Low	High	
**Male**					
	Elevated WC, *n* (%)	398 (54.60)	292 (61.09)	343 (64.96)	0.001
	High BP, *n* (%)	285 (39.09)	212 (44.35)	257 (48.67)	0.003
	Hyperglycemia, *n* (%)	54 (7.41)	51 (10.67)	81 (15.34)	<0.001
	High TG, *n* (%)	379 (51.99)	258 (53.97)	309 (58.52)	0.069
	Low HDL-C, *n* (%)	270 (37.04)	185 (38.70)	225 (42.61)	0.131
	WC, mean±SD, cm	90.64 (9.36)	92.40 (9.44)	92.89 (8.88)	<0.001
	SBP, mean±SD, mmHg	120.05 (15.73)	122.11 (14.14)	123.86 (16.74)	<0.001
	DBP, mean±SD, mmHg	78.41 (11.38)	79.67 (10.06)	81.50 (11.00)	<0.001
	FBG, mean±SD, mmol/L	5.14 (1.13)	5.36 (1.51)	5.48 (1.65)	<0.001
	TG, mean±SD, mmol/L	2.17 (1.75)	2.24 (1.48)	2.37 (2.00)	0.154
	HDL-C, mean±SD, mmol/L	1.13 (0.23)	1.12 (0.24)	1.09 (0.22)	0.023
**Female**					
	Elevated WC, *n* (%)	289 (29.31)	210 (29.33)	51 (28.81)	0.975
	High BP, *n* (%)	198 (20.08)	142 (19.83)	32 (18.08)	0.306
	Hyperglycemia, *n* (%)	38 (3.85)	22 (3.07)	11 (6.21)	0.104
	High TG, *n* (%)	208 (21.10)	145 (20.25)	38 (21.47)	0.334
	Low HDL-C, *n* (%)	99 (10.04)	80 (11.17)	12 (6.78)	0.153
	WC, mean±SD, cm	80.36 (9.42)	80.17 (7.92)	81.62 (9.45)	0.418
	SBP, mean±SD, mmHg	111.88 (15.41)	110.76 (16.19)	116.46 (16.60)	0.014
	DBP, mean±SD, mmHg	72.65 (10.02)	71.81 (10.63)	74.60 (10.66)	0.101
	FBG, mean±SD, mmol/L	4.92 (0.73)	4.98 (0.97)	5.08 (1.47)	0.236
	TG, mean±SD, mmol/L	1.33 (0.85)	1.38 (1.60)	1.47 (0.97)	0.434
	HDL-C, mean±SD, mmol/L	1.35 (0.28)	1.39 (0.28)	1.38 (0.29)	0.196

WC, waist circumference; BP, blood pressure; TG, triglycerides; HDL-C, high-density lipoprotein cholesterol; SBP, systolic blood pressure; DBP, diastolic blood pressure; FBG, fasting blood glucose.

Table S4. Basic characteristics of participants by salt intake level.

Sex	Component/Indicator	Salt intake level		*P* value
		Low	High	
Male				
	Elevated WC, *n* (%)	445 (56.91)	588 (61.70)	0.048
	High BP, *n* (%)	337 (43.09)	417 (43.76)	0.820
	Hyperglycemia, *n* (%)	81 (10.36)	105 (11.02)	0.716
	High TG, *n* (%)	401 (51.28)	545 (57.19)	0.016
	Low HDL-C, *n* (%)	307 (39.26)	373 (39.14)	0.999
	WC, mean±SD, cm	91.36 (9.45)	92.18 (9.14)	0.069
	SBP, mean±SD, mmHg	121.29 (15.37)	122.18 (15.97)	0.238
	DBP, mean±SD, mmHg	79.31 (10.51)	80.03 (11.36)	0.172
	FBG, mean±SD, mmol/L	5.25 (1.30)	5.35 (1.51)	0.134
	TG, mean±SD, mmol/L	2.13 (1.65)	2.35 (1.85)	0.009
	HDL-C, mean±SD, mmol/L	1.11 (0.24)	1.12 (0.22)	0.896
Female				
	Elevated WC, *n* (%)	143 (26.29)	146 (33.03)	0.025
	High BP, *n* (%)	119 (21.88)	79 (17.87)	0.139
	Hyperglycemia, *n* (%)	19 (3.49)	19 (4.30)	0.626
	High TG, *n* (%)	110 (20.22)	98 (22.17)	0.504
	Low HDL-C, *n* (%)	57 (10.48)	42 (9.50)	0.689
	WC, mean±SD, cm	79.70 (8.85)	81.36 (9.49)	0.005
	SBP, mean±SD, mmHg	112.41 (15.69)	111.74 (15.76)	0.508
	DBP, mean±SD, mmHg	73.01 (10.02)	72.27 (10.43)	0.262
	FBG, mean±SD, mmol/L	4.91 (0.67)	4.99 (1.07)	0.164
	TG, mean±SD, mmol/L	1.32 (0.83)	1.39 (1.24)	0.342
	HDL-C, mean±SD, mmol/L	1.35 (0.27)	1.36 (0.29)	0.768

WC, waist circumference; BP, blood pressure; TG, triglycerides; HDL-C, high-density lipoprotein cholesterol; SBP, systolic blood pressure; DBP, diastolic blood pressure; FBG, fasting blood glucose.

Table S5. Association of tea consumption and salt intake on metabolic syndrome and its components.

Variable	Male		Female	
	OR (95% CI)	*P* value	OR (95% CI)	*P* value
**MetS**				
None tea consumption + Low salt intake	1.00 (Reference)		1.00 (Reference)	
Low tea consumption + Low salt intake	1.22 (0.84, 1.76)	0.301	1.09 (0.45, 2.40)	0.840
High tea consumption + Low salt intake	**1.46 (1.02, 2.11)**	**0.040**	1.09 (0.38, 2.69)	0.866
None tea consumption + High salt intake	1.29 (0.94, 1.77)	0.122	1.34 (0.79, 2.27)	0.282
Low tea consumption + High salt intake	1.20 (0.84, 1.71)	0.323	0.88 (0.34, 1.98)	0.768
High tea consumption + High salt intake	**1.83 (1.30, 2.57)**	**<0.001**	1.73 (0.55, 4.58)	0.300
**Elevated WC**				
None tea consumption + Low salt intake	1.00 (Reference)		1.00 (Reference)	
Low tea consumption + Low salt intake	1.19 (0.83, 1.69)	0.342	0.97 (0.54, 1.67)	0.912
High tea consumption + Low salt intake	1.43 (1.00, 2.04)	0.053	1.17 (0.59, 2.23)	0.636
None tea consumption + High salt intake	1.12 (0.83, 1.50)	0.474	**1.42 (1.02, 1.99)**	**0.040**
Low tea consumption + High salt intake	**1.57 (1.11, 2.22)**	**0.011**	1.39 (0.83, 2.30)	0.200
High tea consumption + High salt intake	**1.69 (1.21, 2.37)**	**0.002**	1.37 (0.63, 2.83)	0.405
**High BP**				
None tea consumption + Low salt intake	1.00 (Reference)		1.00 (Reference)	
Low tea consumption + Low salt intake	1.18 (0.82, 1.71)	0.365	0.74 (0.39, 1.37)	0.357
High tea consumption + Low salt intake	1.18 (0.82, 1.71)	0.363	1.34 (0.66, 2.62)	0.407
None tea consumption + High salt intake	1.22 (0.89, 1.68)	0.207	0.81 (0.54, 1.20)	0.293
Low tea consumption + High salt intake	1.04 (0.73, 1.48)	0.831	0.70 (0.36, 1.27)	0.253
High tea consumption + High salt intake	1.28 (0.91, 1.80)	0.155	0.85 (0.34, 1.93)	0.712
**Hyperglycemia**				
None tea consumption + Low salt intake	1.00 (Reference)		1.00 (Reference)	
Low tea consumption + Low salt intake	1.16 (0.61, 2.18)	0.648	2.03 (0.60, 5.96)	0.218
High tea consumption + Low salt intake	**1.82 (1.03, 3.26)**	**0.040**	0.97 (0.14, 4.02)	0.974
None tea consumption + High salt intake	1.28 (0.72, 2.29)	0.399	1.12 (0.45, 2.75)	0.797
Low tea consumption + High salt intake	1.58 (0.88, 2.88)	0.127	2.20 (0.70, 6.19)	0.149
High tea consumption + High salt intake	**1.88 (1.10, 3.29)**	**0.024**	2.07 (0.41, 7.64)	0.313
**High TG**				
None tea consumption + Low salt intake	1.00 (Reference)		1.00 (Reference)	
Low tea consumption + Low salt intake	0.95 (0.67, 1.36)	0.788	1.23 (0.66, 2.20)	0.503
High tea consumption + Low salt intake	0.81 (0.56, 1.15)	0.236	1.79 (0.89, 3.48)	0.092
None tea consumption + High salt intake	1.03 (0.76, 1.40)	0.835	1.34 (0.91, 1.98)	0.132
Low tea consumption + High salt intake	1.02 (0.73, 1.44)	0.894	1.12 (0.61, 1.98)	0.706
High tea consumption + High salt intake	**1.49 (1.06, 2.09)**	**0.020**	1.15 (0.46, 2.60)	0.747
**Low HDL-C**				
None tea consumption + Low salt intake	1.00 (Reference)		1.00 (Reference)	
Low tea consumption + Low salt intake	1.26 (0.88, 1.81)	0.206	0.49 (0.16, 1.20)	0.155

High tea consumption + Low salt intake	1.23 (0.85, 1.76)	0.267	0.86 (0.30, 2.12)	0.763
None tea consumption + High salt intake	1.14 (0.84, 1.56)	0.398	0.96 (0.58, 1.58)	0.882
Low tea consumption + High salt intake	1.04 (0.73, 1.48)	0.823	0.61 (0.24, 1.36)	0.264
High tea consumption + High salt intake	1.29 (0.93, 1.81)	0.133	0.21 (0.01, 1.06)	0.139

The models were adjusted for age, ethnicity, education level, marital status, annual income, shift work, chemical substance exposure, noise exposure, dust exposure, cigarette smoking, alcohol drinking, physical activity, and food diversity. Results in bold indicate statistical significance. OR, odds ratio. CI, confidence interval. MetS, metabolic syndrome; WC, waist circumference. BP, blood pressure. TG, triglycerides. HDL-C, high-density lipoprotein cholesterol.

Table S6. Results of interaction analyses between sex and tea consumption, salt intake, and their combined exposure on metabolic syndrome and its components.

Variable	Tea consumption	Salt intake	Tea and salt combination
MetS	0.737	0.771	0.970
Elevated WC	0.184	0.507	0.465
High BP	0.623	0.087	0.384
Hyperglycemia	0.565	0.832	0.879
High TG	0.782	0.510	0.250
Low HDL-C	0.079	0.592	0.108

MetS, metabolic syndrome; WC, waist circumference. BP, blood pressure. TG, triglycerides. HDL-C, high-density lipoprotein cholesterol. Values presented are *P* values for interaction between sex and each exposure variable (tea consumption, salt intake, and their combination) in relation to metabolic syndrome and its components, based on multivariable logistic regression models adjusted for age, ethnicity, education level, marital status, annual income, shift work, chemical substance exposure, noise exposure, dust exposure, cigarette smoking, alcohol drinking, physical activity, and food diversity.

Table S7. Association of tea consumption and salt intake on metabolic syndrome and its components, after employing imputation for covariates.

Variable	Male		Female	
	OR (95% CI)	*P* value	OR (95% CI)	*P* value
**MetS**				
None tea consumption + Low salt intake	1.00 (Reference)		1.00 (Reference)	
Low tea consumption + Low salt intake	1.15 (0.84, 1.57)	0.382	1.11 (0.53, 2.16)	0.769
High tea consumption + Low salt intake	1.35 (0.96, 1.88)	0.080	1.21 (0.46, 2.84)	0.677
None tea consumption + High salt intake	1.21 (0.91, 1.62)	0.188	1.22 (0.75, 1.97)	0.414
Low tea consumption + High salt intake	1.18 (0.87, 1.61)	0.297	0.81 (0.37, 1.63)	0.569
High tea consumption + High salt intake	**1.80 (1.32, 2.46)**	**<0.001**	1.92 (0.68, 4.73)	0.183

The models were adjusted for age, ethnicity, education level, marital status, annual income, shift work, chemical substance exposure, noise exposure, dust exposure, cigarette smoking, alcohol drinking, physical activity, and food diversity. Results in bold indicate statistical significance. OR, odds ratio. CI, confidence interval. MetS, metabolic syndrome.

Table S8. Association of tea consumption and salt intake on metabolic syndrome and its components, performing analyses based on National Cholesterol Education Program Adult Treatment Panel III criteria for MetS.

Variable	Male		Female	
	OR (95% CI)	*P* value	OR (95% CI)	*P* value
**MetS**				
None tea consumption + Low salt intake	1.00 (Reference)		1.00 (Reference)	
Low tea consumption + Low salt intake	1.29 (0.89, 1.86)	0.173	0.89 (0.46, 1.64)	0.721
High tea consumption + Low salt intake	**1.49 (1.04, 2.14)**	**0.031**	1.68 (0.84, 3.26)	0.129
None tea consumption + High salt intake	1.27 (0.92, 1.74)	0.142	1.18 (0.80, 1.73)	0.406
Low tea consumption + High salt intake	1.20 (0.84, 1.72)	0.305	0.89 (0.47, 1.61)	0.719
High tea consumption + High salt intake	**1.92 (1.37, 2.69)**	**<0.001**	1.44 (0.63, 3.10)	0.364

The models were adjusted for age, ethnicity, education level, marital status, annual income, shift work, chemical substance exposure, noise exposure, dust exposure, cigarette smoking, alcohol drinking, physical activity, and food diversity. Results in bold indicate statistical significance. OR, odds ratio. CI, confidence interval. MetS, metabolic syndrome.

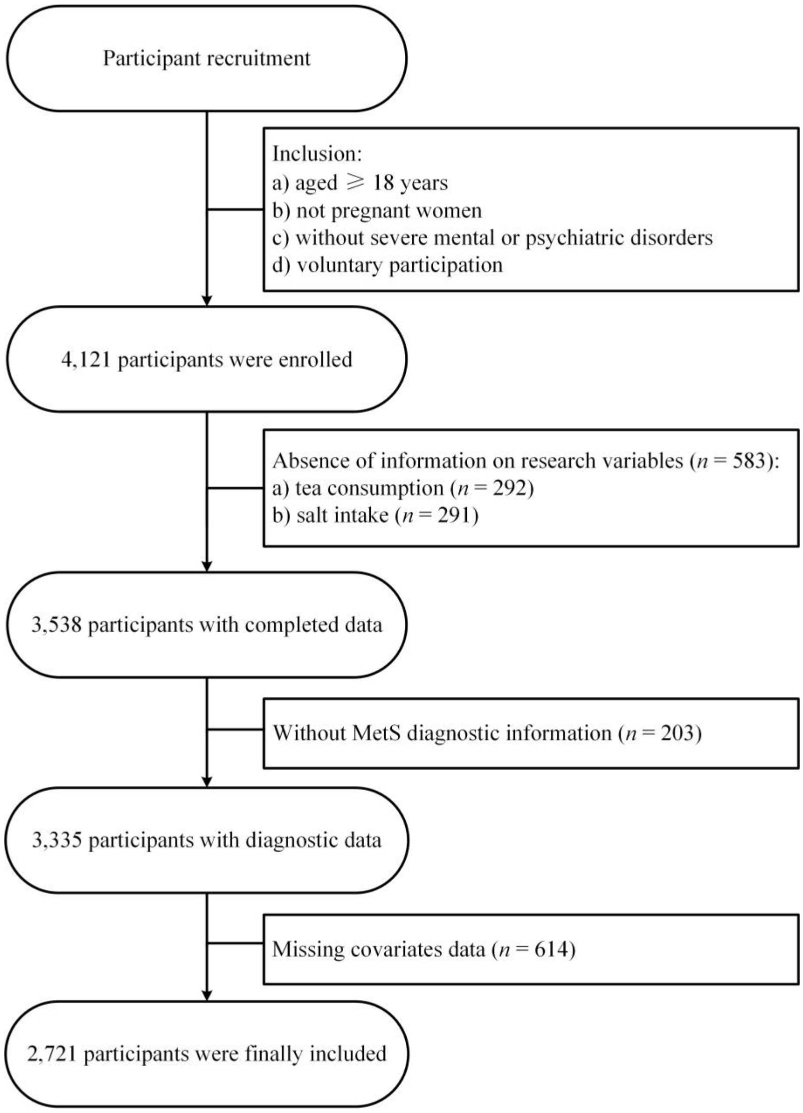

Figure S1. Flow diagram of participants included in this study.
